# Investigating the risk factors for academic difficulties in the medical programme at a South African university

**DOI:** 10.1186/s12909-022-03274-1

**Published:** 2022-03-28

**Authors:** Sfiso Emmanuel Mabizela, Judith Bruce

**Affiliations:** 1 Centre for Health Science Education, Faculty of Health Sciences, University of the Witwatersrand, Johannesburg, South Africa; 2 School of Therapeutic Sciences, Faculty of Health Sciences, University of the Witwatersrand, Johannesburg, South Africa

**Keywords:** Risk factors, Academic difficulties, NBT, Medical students

## Abstract

**Background:**

The National Benchmark Test (NBT) that determines academic readiness is widely used by Faculties as an additional measure to select students for the study of medicine. Despite this, many students continue to experience academic challenges that culminate in delayed graduation and sometimes academic exclusion or discontinuation of studies.

**Aim:**

This study aimed to understand academic and non-academic variables linked with academic difficulties in the first three years of medical education.

**Methods:**

The study sample consisted of six cohorts of medical students for the period 2011 to 2016 (*n* = 1392). Only the first three of the six-year medical programme were selected for analysis. Survival analysis and Cox Proportional Hazard (CPH) was used to identify academic and non-academic variables associated with academic difficulties.

**Results:**

A total of 475 students (34%) experienced academic difficulty; 221 (16%) in the first year of study, 192 (14%) in the second year and 62 (5%) in the third year of study. The results show that Intermediate Upper, Lower and Basic levels for all NBT domains, living in university residence, rurality and male gender were risk factors for academic difficulty.

**Conclusion:**

In mitigating these factors, the NBT must inform the type of support programmes to augment the students' skills and promote academic success. Additionally, existing support programmes should be evaluated to ascertain if they reach students at risk and whether participating in these programmes yield positive academic outcomes.

## Introduction

Faculties of Health Sciences, historically known as medical schools in South Africa, are under increasing pressure to produce more doctors to address the perceived shortages arising from the misdistribution of doctors between urban and rural settings [1] and the growing trend among doctors who emigrate in search of work in developed countries [2]. In addressing these shortages, medical student admission has been increased [3]; however, higher admission rates do not translate into higher throughput rates [2]. Countrywide, approximately 1200 doctors graduate annually; this figure exacerbates the ratio of fewer than one doctor per 1000 population between 2010 (0.72) and 2017 (0.91), respectively [4]. Furthermore, a higher dropout rate is notable as 40.2% and 22.7% of first-time students who enrolled for a six-year undergraduate degree discontinued their studies between 2000 and 2012 [5]. The National Senior Certificate (NSC) alone is not sufficient to determine students' levels of preparedness for university, creating a need for an additional selection tool [6].

In 2005, the University of South Africa commissioned the NBT to assess applicants’ readiness for higher education. The NBT assesses students’ readiness in NBT Mathematics (NBT MAT), NBT Academic literacy (NBT AL) and NBT Quantitative Literacy (NBT QL). The NBT MAT is a Mathematics test designed to assess students’ knowledge and understanding [7]. The NBT AL assesses students’ ability to engage successfully with the language demands of higher education; NBT QL assesses students’ ability to engage with the quantitative demands of higher education [8, 9], which includes their problem management/solving ability through the use of quantitative information in all its formats. The premise for using the NBT arises from the weak predictive ability of the NSC to determine academic success in higher education [6, 10].

The NBT is a criterion-referenced test, that is, it assesses students’ capacities in clearly defined domains to determine their levels of competency and for placement in appropriate curriculum routes [8, 9]. The NBT has three benchmark levels for student performance: Proficient, Intermediate and Basic. A Proficient score suggests that students are likely to cope with the academic demands of the programme. Although such a student might fail, it is not likely to be related to the domains tested [7]. Divided into two levels, the Intermediate Upper level highlights the expectation of learning challenges. Students with scores in the Intermediate Upper level would likely need additional academic support and special skills training, and scores in the Intermediate Lower level indicate that students would likely need placement in an extended programme [7]. Basic performance levels suggest that students are not prepared for higher education study and will not cope if admitted for a degree programme in the absence of long-term support, such as a foundation programme [7]. These performance descriptors provide institutions with information about students’ entry-level skills and how they should respond to incoming students.

Characteristic of the era, the university in this study, inherited the legacy of apartheid, which influenced decisions on access to medical school [11]. As a result, racialised throughput patterns are still being observed [9]. In response to issues of inequitable access, the Faculty under study adjusted its admission requirements to facilitate admission of students who are perceived to have the potential to cope well with the academic demands of the medical programme. The revised admission policy widened entrance for qualifying students in three additional categories not previously considered. In the new admission policy, 40% of places in the medical programme are reserved for top-performing students, and 60% for top-performing applicants from rural areas, quintile 1 and 2 (under-resourced) schools and those classified as Black and Coloured. In particular, an increase in the number of students from rural areas from 2015 onwards could be attributable to the adjustment in the admission policy.

Until five years ago, five out of nine Faculty of Health Sciences/medical schools in South Africa used the NSC and the NBT to select students for the medical programmes [3, 12, 13]. These medical schools use different weightings of these two components to select students, and the compulsory NSC subjects are required at specified levels of achievements [3]. At the study institution, both the NBT and the NSC contribute 50% to an applicant’s composite index [8]. The NBT performance descriptors provide institutions with information about students’ entry-level skills and in turn, how they should respond to incoming students [9]. Meaningful in this regard is how institutions manage the academic profile of their incoming students. Given the reality of an excessive number of applications relative to available places in medical schools, and if an institution preferentially accepts applicants with Proficient NBT scores, it is likely to maintain a profile of historical advantage in its student body. To avoid this ‘pitfall’, the new admission policy introduced modifiers that aimed to identify and admit disadvantaged students with the potential to succeed. For example, employing this methodology would offer a Black applicant from a rural quintile 1 school and an intermediate NBT level the same opportunity as an urban, white NBT-Proficient applicant from a private school. This potentially introduces risk for both the student and the institution that must be managed by paying close attention to at-risk students’ progress and support requirements.

## Literature review

Multiple studies suggest that medical students' academic difficulties tend to occur in the early years of training, resulting in an early exit from the programme [14–17]. Personal issues, performance in admission tests, learning environments, and the curriculum structure are some of the variables associated with academic difficulties [14, 15]. Furthermore, medical students may fail to identify their own learning challenges and not seek help [16, 18, 19]. The underlying variables that predict academic difficulties are complicated, and they need to be explored continuously to inform support programmes and improve retention. Variables such as Grade Point Average (GPA), socio-economic status, gender, attitudes and progress performance are some of the variables that have been used in prediction models [20–22].

The consequences of academic difficulties result in the protracted time to graduation and a high rate of attrition that have implications for students, educators and institutions in terms of lost resources and opportunities [20]. For the students, failing may produce a variety of psychological issues such as lower levels of self-efficacy, depression, and low self-esteem, burnout, mental health issues and social isolation [23–25]. The consequence for universities is the possibility of losing government subsidies in an already underfunded higher education sector [26, 27].

Global higher education institutions are affected by low throughput rates (Prince, 2016). In South Africa, most students need additional years to complete their degrees, while others discontinue their studies altogether [10, 27, 28]. Some students may discontinue their studies due to the costs associated with higher education [26]. On the other hand, financial need may exacerbate academic exclusion possibilities as students tend to redirect the funds they receive to support their families [27]. The school quintile system contributes to students’ low throughput and lack of preparation for higher education [29, 30]. Socio-economic indicators are central in classifying the five school quintiles (SQ), with quintile one (Q1) schools considered the poorest and quintile five (Q5), the most affluent, well-resourced schools. Furthermore, teachers with substantial teaching knowledge are saturated in Q5 schools [30], which is not the case in lower quintile schools.

Student admission data are an essential resource to identify students who may struggle academically [21]. Academic markers such as the NBT and the NSC and non-academic variables should be utilised to determine students' success and academic difficulties. There is a scarcity of studies addressing predictors of academic difficulties in South African Faculties of Health Sciences, despite the high rate of delayed graduations and dropouts [8]. Considering that academic difficulties tend to manifest in the early years of a medical programme, this study investigated the predictors of academic difficulties in the first three years of study. The medical curriculum comprises two preclinical years and four clinical years [13], with the introduction of the basic sciences during the first two years [31]. Medical students begin clinical training in the third and fourth year, and the fifth and sixth are full clinical years in which the students are placed in clinical clerkships in academic hospitals and in community and rural sites [31].

## Study aim

This study aimed to investigate the predictors for academic difficulties in the first three years of the medical programme. The objectives were to determine the probability of survival in the first three years of the medical programme and examine the risk of experiencing academic difficulty in respect of academic and non-academic variables.

## Methods

This study was conducted at the Faculty of Health Sciences of the University of the Witwatersrand. A purposively selected sample consisted of 1392 first-time medical students registered between 2011 and 2016. After processing and cleaning the data, 15 missing variables were removed from the dataset, resulting in 1377 cases for analysis (*n* = 1377).

The data, obtained from the University’s Business Intelligence Service (BIS), comprised non-academic and academic variables. Non-academic variables included gender, race, place of origin, residence and school quintile attended; academic variables were the NBT results and students’ progression outcome for the first three years of the medical programme. The data was reviewed during the process of cleaning and preparing for analysis. No further reviews were made after the analysis was completed. The progression outcome explicitly specifies whether the student was permitted to proceed, cancelled or had to repeat a year. Academic difficulty in this study captures students who repeated, dropped out or supplemented any of the first three years of study. Other than term withdrawal, cancellation of studies and failing to meet readmission committee conditions, the data does not provide more information of students who have discontinued their studies.

Ethical approval to conduct the study was obtained from the University’s Human Research Ethics Committee (Medical) (HREC M170490).

### Data analysis

A large data set that was 99% complete was cleaned and prepared for analysis. Survival analysis and Cox Proportional-Hazards (CPH) models were used to analyse the data. The survival analysis models were used to compare the profiles of students who experienced academic difficulties in the first three years of study using the academic variables (NBT domains proficiency levels) and non-academic variables (gender, place of origin, school quintile and residence). Students who did not experience academic difficulties for the entire observation period were excluded. Kaplan–Meier estimates were used to calculate the probability of survival in the medical programme in the first three years. The survival plots were used to compare survival distribution between groups, and the log-rank tests determined whether survival curves were statistically significant between the groups of students who experienced academic difficulties [32].

The CPH was used to investigate the risk factors for academic difficulties. The CPH model is a semi-parametric type of analysis that measures observed covariates' effects on the event's risk [33]. The CPH assumes that the likelihood of the event occurring is mediated by the linear combination of covariates referred to as linear proportional hazard [33, 34].

### Description of data labelling

The NBT performance levels for all domains were categorised into two binary variables. The students who achieved proficiency in all the NBT results were separated from the students who achieved results that placed them at Intermediate Upper, Lower and Basic performance levels: NBT MA, Proficient = 0, Intermediate Upper, Lower and basic = 1; NBT AL, Proficient = 0, Intermediate Upper, Lower and basic = 1; NBT QL, Proficient = 0, Intermediate Upper, Lower and basic = 1; Place of origin, Urban = 0, Rural and Unknown = 1; Gender, Male = 0, Female = 1); Living arrangements, (Living at the university residence = 0, Living in private residence = 1). School quintile, SQ5 = 0, SQ1-4 = 1.

The regression coefficient of explanatory variables has either positive or negative (B) values that suggest the relationship and the magnitude between variables. If a positive B value is greater than 1, it suggests higher risks for academic difficulty for the group coded 1 than 0. If the B value equals zero, it indicates no difference in the two groups' risk of experiencing academic difficulty. If the B value is less than zero, it suggests that the risk of academic challenges is higher for the group coded 0 than the group coded 1.

The Exp(B) shows the hazard ratio and the extent to which the hazard ratio is lower or higher for the groups under study [35]. The hazard ratio with positive values suggests higher risks of academic difficulty for the group coded 1, and a negative value indicates that the group coded 0 has higher risks of academic difficulty.

## Results

### Student demographics

The racial breakdown showed that 44% of the sample (*n* = 605) were reportedly Black; 1% (*n* = 11) were Chinese, 6% (*n* = 80) were Coloured, 27% (*n* = 375) were White, and 22% (*n* = 306) were Indian. The gender profile depicted that 585 (42.5%) were male, and 792 (57.5%) were female. In terms of place of origin, 1090 (79%) were of urban origin, 218 (16%) emanated from rural areas, and 69 (5%) did not indicate their place of origin. A total of 922 (67%) students lived off-campus, while 455 (33%) lived in various university residences. The number of off-campus students may well include those away from home or from rural areas, but the data were not sufficiently differentiated to determine this. Table 1 shows the demographics of the sample by the selected cohorts. A total of 475 (34.5%) of students experienced academic difficulty: 221 (16%) in the first year of study, 192 (14%) in the second year and 62 (4.5%) in the third year of study; 902 (65.5%) did not experience academic difficulty in the first three years of study.

**Table 1:** Demographic profile of the sample (*n* = 1377)

**Variables**	**2011**	**2012**	**2013**	**2014**	**2015**	**2016**	**Total %**
**Race**							
**Black**	70 (38%)	130 (48%)	103 (47%)	99 (40%)	97 (40%)	106 (49%)	44%
**Chinese**	3 (2%)	3 (1%)	1 (1%)	2 (1%)	2 (2%)	0	1%
**Coloured**	14 (8%)	11 (4%)	8 (4%)	17 (7%)	18 (7%)	12 (5%)	6%
**Indian**	42 (23%)	45 (16%)	43 (19%)	65 (27%)	56 (23%)	55 (25%)	22%
**White**	52 (29%)	84 (31%)	65 (29%)	61 (25%)	68 (28%)	45 (21%)	27%
**Gender**							
**Female**	114 (63%)	175 (64%)	127 (57%)	141 (58%)	128 (49%)	107 (57, 5%)	58%
**Male**	67 (37%)	98 (36%)	93 (42%)	103 (42%)	113 (51%)	111 (42, 5%)	42%
**Place of origin**							
**Rural**	14 (8%)	22 (8%)	23 (11%)	27 (11%)	66 (27%)	66 (30%)	16%
**Unknown**	20 (11%)	20 (7%)	7 (3%)	6 (3%)	11 (5%)	5 (5%)	5%
**Urban**	147 (81%)	231 (85%)	190 (86%)	211 (86%)	164 (68%)	147 (67%)	79%
**Residence**							
**Off campus**	170 (94%)	217 (79, 5%)	136 (62%)	157 (64%)	116 (48%)	126 (58%)	67%
**University residences**	11 (6%)	56 (20, 5%)	84 (38%)	87 (36%)	125 (52%)	92 (42%)	33%

### Cox proportional regression results

The Omnibus Test of Model (OTM) coefficient confirmed the overall predictive fit of the model. The Chi-square results were statistically significant: χ^2^ (7, *N* = 1377) 100.902,* p* < 0.000, which confirms that the model was a significant fit relative to the null hypothesis.

After considering all variables in the equation, males *(p* < 0.004) and students living in university residence (*p* < 0.005) were statistically significant risk factors for academic difficulties. Male and students living in university residences exhibited greater hazards for experiencing academic difficulties, 24.6% and 30.4%, respectively. Place of origin (*p* = 0.126) and school quintiles (*p* = 0.267) were not statistically significant in students experiencing academic difficulty. However, students of rural origin showed greater for experiencing academic difficulties with 20.4% hazards ratios compared to students of urban origin. Likewise, the students who attended Q1 to Q4 schools also showed greater hazards (14.7%) than students who attended Q5 schools.

The results for academic variables show that the NBT MA (*p* < 0.000) and NBT QL (*p* < 0.000) were statistically significant risk factors for academic difficulty. The students with Intermediate Upper, Lower and Basic levels had lower survival time in the medical programme. The hazard ratios were 76.8% for the NBT MAT and 53.8% for the NBT QL, suggesting higher risks of academic difficulties in the first three years of the medical programme. The NBT AL domain was not statistically significant (*p* = 0.610). See Table 2.

**Table 2:** Risk ratios for non-academic and academic variables

***Variables***	B [C1 95%]	*df*	Exp(B)	Sig
*Gender*	-.282 [.622, .915]	1	.754	.004
*Place of origin*	.186 [.949, 1.527]	1	1.204	.126
*Residence*	.265 [1.082, 1.571]	1	1.304	.005
*School Quintile*	.136 [.899, 1.461]	1	1.147	.267
*NBT Mathematics*	.570 [1.433, 2.182]	1	1.768	.000
*NBT Academic literacy*	-.062 [.742, 1.192]	1	.941	.610
*NBT Quantitative Literacy*	.431 [1.237, 1.913]	1	1.538	.000

### Survival analysis results

**Fig. 1:**
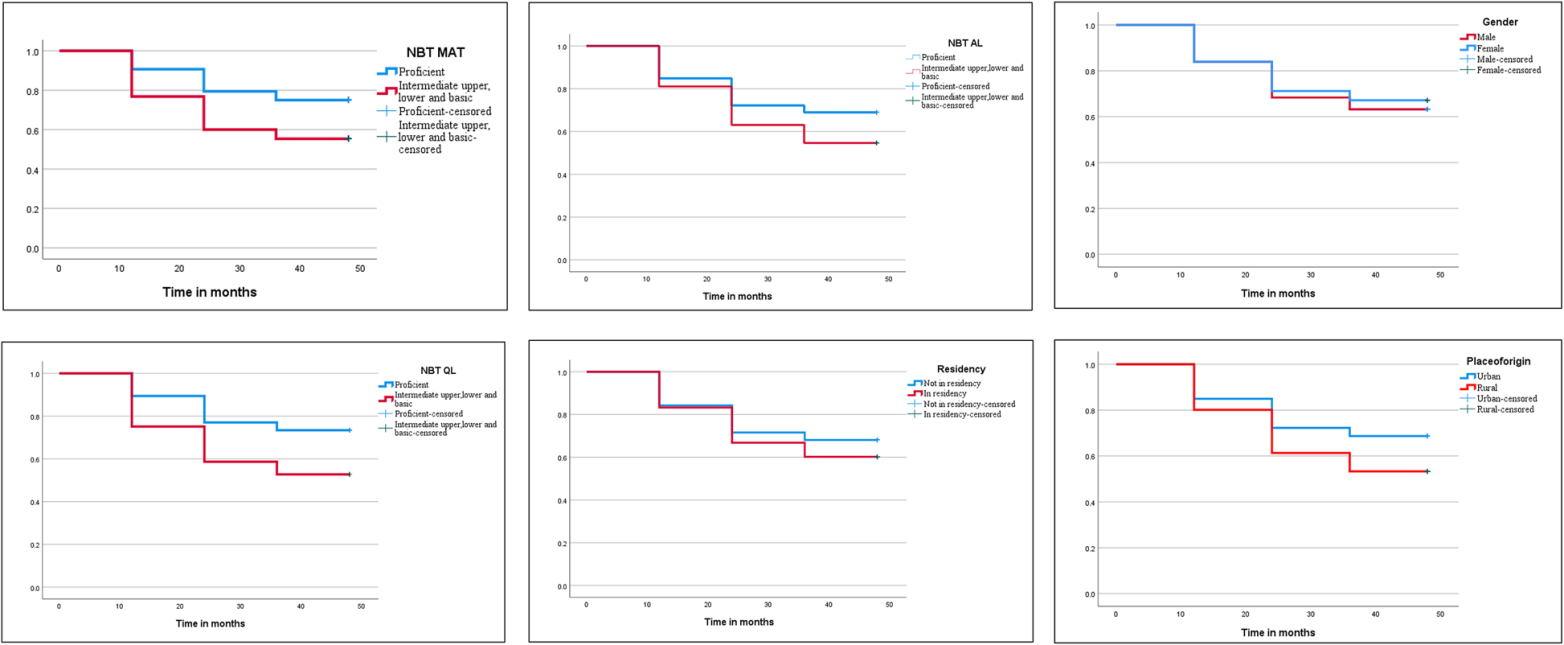
Survival plots for the NBT domains, gender, place of origin and residency

The survival distribution for NBT MA was statistically significant (χ^2^ (1) = 63.648, *p* < 0.000). The sharp drop in the survival plot (Fig. 1) indicates the first, second and third year of study where 93 (13.1%) and 84 (11.8%), and 33 (4.7%) students with a proficiency level in the NBTMA, experienced academic difficulties. In the Intermediate Upper, Lower and Basic level of the NBT MA, 128 (19.2%), 108 (16.2%), and 29 (4.3%) students also encountered academic difficulties in the first three years. Of the 475 students who failed, 210 (44%) obtained a Proficient level, and 265 (56%) obtained Intermediate Upper, Lower and Basic levels in the NBT MA.

The overall survival distribution for NBT AL was statistically significant (χ^2^ (1) = 21,165 *p* < 0.000). The risk of experiencing academic difficulty was higher in the second and third year of study for students with Intermediate Upper, Lower and Basic levels (47 (14.1%) and 22 (6.6%) respectively) than students with Proficiency level (145 (13.9%) and 40 (3.8%) respectively). Of the 475 students who failed, 350 (74%) obtained Proficiency levels, and 125 (26%) obtained Intermediate Upper, Lower and Basic levels in the NBT AL.

The survival distribution for NBT QL was statistically significant (χ^2^ (1) = 67.013, *p* < 0.000). A similar pattern of more students experiencing academic difficulties in the first two years was observed. The first two sharp drops show higher risks for students with Intermediate Upper, Lower and Basic levels: 109 (20.7) and 85 (16.1%), than students with proficiency levels: 112 (13.2%) and 107 (12.6%) in the first two years of study. Of the 475 students who failed, 251 (53%) had a Proficiency level, and 224 (47%) had Intermediate Upper, Lower and Basic levels in the NBT QL.

Survival distribution for gender yielded no statistically significant results (χ^2^ (1) = 1.945, *p* < 0.163). Of the 475 who experienced academic difficulties, 201 (42%) were male, and 274 (58%) were female students. The survival function results comparing students who lived in a university residence with those who live off-campus were statistically significant (χ^2^ (1) = 7.298, *p* < 0.007). Overall, 313 (66%) who live in private residential and 162 (34%) who live in university residences experienced academic challenges. Students' survival by place of origin was statistically significant (χ^2^ (1) = 22.763, *p* < 0.00). Based on the survival plots, students of rural origin are at a higher risk for experiencing academic difficulties.

## Discussion

This study focused on the first three years of the medical programme using academic and non-academic variables to determine their predictive value in students’ experiencing academic difficulties. Intermediate Upper, Lower and Basic levels in any of the three NBT domains were linked with future academic difficulties for students early on in their medical degree studies. The hazards ratios for NBTMA and NBTQL suggest more serious academic difficulties for the students admitted with Intermediate Upper, Lower and Basic levels. Also, being male, living in a university residence and being of rural origin were significant risk factors for academic difficulties.

Several studies on medical students report that more students encounter academic difficulties in the early years than in the later years of medical education [14–17]. Though limited to the first three years, this study highlights those variables linked with early academic challenges in the medical programme and provides an early warning system for which decisive interventions are required.

The NBT test states that students with Intermediate Upper levels require complementary support in tutorials, workshops, augmented courses and language while students with Intermediate Lower results should be placed in a foundation programme, and the students with a Basic level have learning challenges [7]. This study suggests the importance of support for students admitted with Intermediate Upper, Lower and Basic levels as they are at higher risk for academic difficulties than students with Proficiency levels. The higher risk of academic challenges for these students may also suggest a lack of adequate preparation for academic programmes [26, 31].

Students of rural origin were more prone to encounter academic difficulties than students of urban origin. In a study exploring academic challenges in an Australian regionally located school, the Australian Standard Geographical Classification-Remoteness Area (ASGC-RA), a rural area, was a significant predictor of academic difficulty [34]. Students who attend lower quintile schools in rural areas tend to receive substandard education in the South African context as teachers with substantial subject knowledge are concentrated in higher quintile schools [31, 36]. Also, disparities in socio-economic status may produce cultural capitals detrimental to students' academic progress from rural and lower quintile schools [37]. While access and financial assistance may be available, rural students usually lack epistemological access to navigate university spaces [27]. As observed in this study, 38% of students of rural origin encountered academic challenges. Therefore, it is incumbent upon universities to support and assist students in adapting to the university's socio-cultural life.

Gender was one of the significant predictors of academic difficulties. More specifically, male students are at a higher risk than their female counterparts. A South African study investigating medical students’ perceptions of factors affecting their academic performance found that 61% of male students perform poorly [17]. The central concern about male students is that they are less likely to seek help despite poor performance indicators [18]. However, observed no difference in the academic performance between male and female medical students’ cumulative GPA. The most critical issue, thus, is that the university should be proactive in identifying underperforming male students early on and offer essential support to engender positive academic outcomes.

## Limitations and implications for future studies

There are many reasons why some students may discontinue their studies other than academic difficulties. Variables such as parents' marital status, first-generation students, financial issues, and career changes were not considered. Also, the data set received from the university’s BIS unit does not explain in detail why a student discontinues the programme. There is a need for research to uncover reasons for discontinuing. This study focused only on the first three years of study, where we observed that fewer students were experiencing academic challenges in the third year. It would be essential to understand how these students’ progressed to the sixth year of study. Furthermore, the study was conducted in a single university, and the authors acknowledge that the results would be different if more universities that used similar entry requirements were included. Graduate entrants who start in the third year of the medical programme were excluded from this study.

## Conclusion

This study found that students with Intermediate Upper, Lower and Basic levels in any of the three NBT domains are at higher risk of encountering academic difficulties. Based on the survival analysis NBTMA and NBTQL are significant predictors of academic difficulty during the first three years of study. Male students, students who live in university residences and students who are from rural areas are at a significantly higher risk of experiencing academic difficulties. In the interest of social accountability, the NBT scores must inform the type of support programme to augment the students' skills and promote their chances of academic success. It is recommended that students admitted with scores that place them in Intermediate Upper and Lower levels be considered for tailored academic support interventions – these may vary from complementary tutorials to foundations programmes and extended curricula. Further research is required to evaluate the nature of specific support programmes that are available and whether participating in these programmes yield positive outcomes for medical students. In the context of their traditions, the learning culture in students residences needs to be explored and strengthened for such spaces to make positive contributions to student survival and success, focusing on male students in medicine and students of rural origin. We recommend that current faculty interventions targeting rural students be more intentional in including medical students and be sustained over several semesters for a more significant impact. Knowledge of survival probabilities and predictors of academic difficulties leads to early identification of at-risk students and the design of interventions to improve their chances of succeeding in their studies.

## Data Availability

The data used in this study are available from the corresponding author subject to ethical approval.

## Abbreviations

NBT: National Benchmark Test
NBTMA: National Benchmark Test Mathematics
NBTAL: National Benchmark Test Academic Literacy
NBTQL: National Benchmark Test Quantitative Literacy
NSC: National Senior Certificate
SQ: School Quintile
CPR: Cox Proportional Hazard
BIS: Business Intelligence Service
OTM: Omnibus Test of Model
